# Golden Gate Shuffling: A One-Pot DNA Shuffling Method Based on Type IIs Restriction Enzymes

**DOI:** 10.1371/journal.pone.0005553

**Published:** 2009-05-14

**Authors:** Carola Engler, Ramona Gruetzner, Romy Kandzia, Sylvestre Marillonnet

**Affiliations:** Icon Genetics GmbH, Biozentrum Halle, Halle, Germany; Virginia Tech, United States of America

## Abstract

We have developed a protocol to assemble in one step and one tube at least nine separate DNA fragments together into an acceptor vector, with 90% of recombinant clones obtained containing the desired construct. This protocol is based on the use of type IIs restriction enzymes and is performed by simply subjecting a mix of 10 undigested input plasmids (nine insert plasmids and the acceptor vector) to a restriction-ligation and transforming the resulting mix in competent cells. The efficiency of this protocol allows generating libraries of recombinant genes by combining in one reaction several fragment sets prepared from different parental templates. As an example, we have applied this strategy for shuffling of trypsinogen from three parental templates (bovine cationic trypsinogen, bovine anionic trypsinogen and human cationic trypsinogen) each divided in 9 separate modules. We show that one round of shuffling using the 27 trypsinogen entry plasmids can easily produce the 19,683 different possible combinations in one single restriction-ligation and that expression screening of a subset of the library allows identification of variants that can lead to higher expression levels of trypsin activity. This protocol, that we call ‘Golden Gate shuffling’, is robust, simple and efficient, can be performed with templates that have no homology, and can be combined with other shuffling protocols in order to introduce any variation in any part of a given gene.

## Introduction

Current protocols for assembling variant gene libraries have evolved from the relatively simple early protocols that generated random variability through error prone PCR [1] into a rich variety of protocols that allows introduction of virtually any type of variation in any given gene [2], [3], [4]. For example, libraries can be constructed from pools of DNaseI digested fragments prepared from parental templates [5], [6], [7], [8], from degenerate oligonucleotides [9], [10] or from mixtures of both, or even from undigested parental templates [11], [12], [13], and are usually assembled through PCR. Libraries can also be made from parental sequences recombined *in vivo* or *in vitro* by either homologous or non-homologous recombination [14], [15], [16].

Despite the large diversity of existing DNA shuffling protocols, standard cloning methods based on restriction enzymes are not widely used in these protocols. One obvious reason is that current cloning methods are usually not efficient enough to generate the large number of variants required for DNA shuffling. Using restriction enzymes would have several advantages such as providing the ability to shuffle genes irrespective of their degree of homology, providing flexibility and control regarding the number of recombination events in each shuffled gene, and the ability to shuffle very large genes or several regions within large genes (independence from PCR amplification). In fact, two DNA shuffling strategies have earlier been developed based on the use of type IIB or type IIs restriction enzymes [17], [18], [19], [20]. However, these protocols are quite complex to perform, require several successive steps, and in many cases still rely on PCR for amplification of the library since only small amount of recombinant templates is obtained.

We have recently developed a protocol that allows subcloning a DNA fragment from one plasmid to another with very high efficiency in one tube and one step [21]. This protocol is also based on the use of type IIs restriction enzymes, and allows the conversion of more than half of all input plasmids into the desired recombinant product in just a 30 minutes restriction-ligation. High efficiency was also reported for the cloning of one to three PCR products using a similar cloning strategy [22]. We have now developed a protocol for cloning multiple fragments at once, and show here that at least 9 different fragments can be assembled together in a defined linear order and inserted into a recipient plasmid in one step, and that such procedure is so efficient that the majority (about 90%) of colonies growing on selection plates contain the desired constructs. This efficiency is sufficient to generate libraries of recombinant genes from several parental templates.

We have used trypsinogen as a test protein for this shuffling protocol. In plants, trypsinogen (bovine cationic trypsinogen) is expressed only at a low level ([23], and our unpublished results), and we suspect that this is due to instability of the protein. We have shuffled trypsinogen together with the genes for bovine anionic and human cationic trypsinogen. Screening of just 225 recombinant clones by transient expression in *Nicotiana benthamiana* leaves led to selection of variants that allows production of a higher amount of trypsin activity per gram of leaf tissue.

## Results

### DNA shuffling strategy

**Figure 1:**
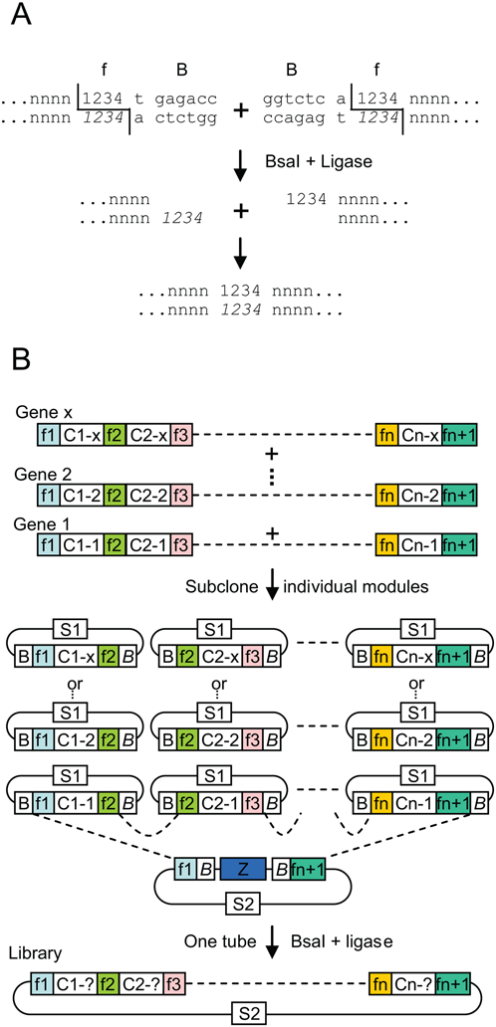
DNA shuffling strategy. (A) Two DNA ends terminated by the same 4 nucleotides (sequence f, composed of nucleotides 1234, complementary nucleotides noted in italics) flanked by a BsaI recognition sequence, B, form two complementary DNA overhangs after digestion with BsaI. (B) For shuffling, genes of interest are aligned, and recombination points consisting of 4 nucleotide sequences (f1 to fn+1) are defined on conserved sequences. Module fragments (core sequence, C1 to Cn, plus flanking 4 nucleotide sequences) are amplified by PCR and cloned in an intermediate cloning vector. Module fragment plasmids and the acceptor vector are assembled in one restriction-ligation with BsaI and ligase. S1 and S2, two different selectable markers. Z, lacZ alpha gene fragment.

In an earlier work, we have shown that a DNA fragment of interest can be subcloned with very high efficiency in one step and one tube from one plasmid to another [21]. The principle of the cloning strategy is based on the ability of type IIs restriction enzymes to cut outside of their recognition site. Two DNA ends can be designed to be flanked by a type IIs restriction site such that digestion of the fragments removes the enzyme recognition sites and generates ends with complementary 4 nt overhangs; such ends can be ligated seamlessly, creating a junction that lacks the original site (Fig. 1A). This property allows cloning to be performed using a one-step restriction-ligation. This strategy was shown to result in the conversion of more than half of all input plasmids present into the desired recombinant product in just a 30 minutes restriction-ligation. Subcloning was also found to be very efficient when two and three inserts were subcloned, but the total amount of recombined plasmid was lower.

This cloning strategy could also be used for DNA shuffling if the entry modules that are subjected to restriction-ligation are prepared from a set of homologous genes rather than from a single gene. Such a DNA shuffling protocol would consist of first selecting a number of 4 nucleotides ‘recombination sites’ (sequence f1 to fn+1, Fig. 1B) on a nucleotide sequence alignment of several homologous genes. Recombination sites would be chosen on sequences that are identical among all homologues, but different from all other selected sites within the same gene. The selection of these recombination sites defines modules that consist of a core sequence (C, sequence variable among homologues) flanked by two 4 nt sequences (f). These modules can be amplified by PCR with primers designed to add flanking BsaI sites on each side of the modules (the BsaI cleavage sites perfectly overlapping with the recombination sites), and cloned in an intermediate cloning vector and sequenced. A restriction-ligation performed on a mix containing all intermediate plasmids (total number of plasmids: x multipled by n), the recipient acceptor vector, BsaI enzyme and ligase is expected to allow assembly of a library of shuffled genes. This is because each module is compatible and can be ligated only to a module belonging to the next consecutive set of homologous modules, or to the acceptor vector for the first and last modules, and because each module from a set of homologous modules can be ligated with equal probability to each module of a contiguous set. In addition, because of the restriction-ligation, only the desired assembled products are expected to accumulate since all other ligation products (for example, ligated products containing plasmid backbone DNA from the intermediate constructs) will contain BsaI sites and should therefore be immediately redigested with BsaI.

As a first step toward testing this protocol, we decided to try to assemble a plasmid from 10 separate input plasmids (9 module plasmids and one vector plasmid) in a restriction-ligation.

### One-pot one-step assembly of a GFP construct from 10 constructs

**Figure 2:**
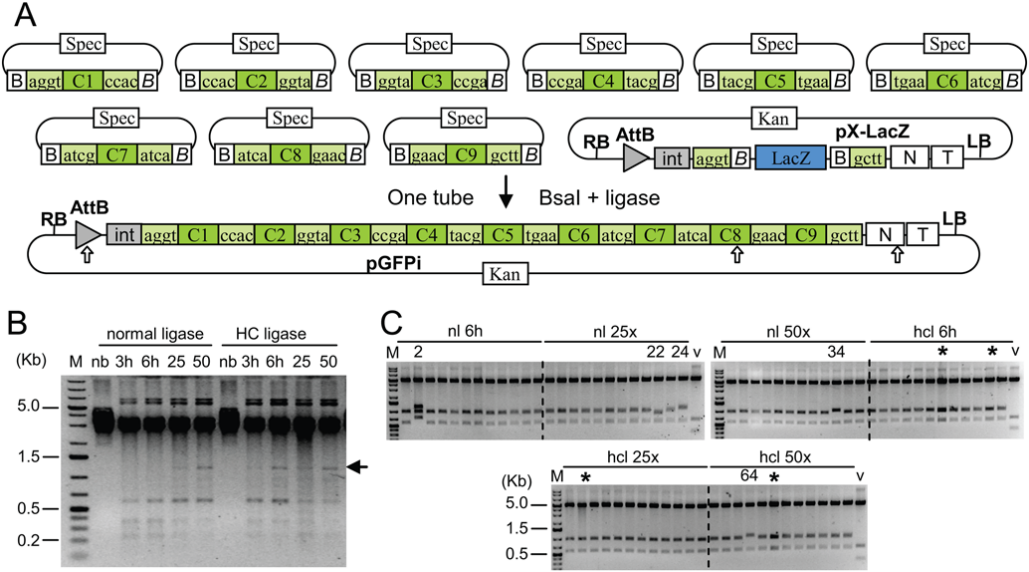
Assembly of a GFP construct from 10 plasmids. (A) Construct maps. Input modules contain a core region C flanked by BsaI restriction sites in opposite orientation composed of a recognition site (B, ggtctcn, *B*, ngagacc) and a 4 nucleotide cleavage site (boxes flanking the core region). pX-LacZ, acceptor vector. pGFPi, resulting construct. Restriction sites for AvrII and XmaI are shown as white arrows. (B) Ethidium bromide-stained gel with products obtained by restriction-ligation of the 9 input module plasmids. M: GeneRuler 1kb DNA Ladder Plus from Fermentas. Restriction-ligation was performed at 37°C for 3 (lane 3h) or 6 hours (lane 6h) or with 25 cycles (2 min 37°C+5 min 16°C, lane 25) or 50 cycles (lane 50), and without BsaI enzyme (lane nb). The arrow indicates the 1.17 kb linear assembled GFP gene product. (C) Ethidium bromide-stained gels of 72 minipreps digested with XmaI and AvrII (expected fragment sizes: 4.6 kb, 945 and 555 bp), obtained from restriction-ligations performed for 6 h 37°C (6 h), for 25 or 50 cycles (25×/50×), with normal ligase (nl) or high concentration ligase (hcl). Numbers indicate minipreps with an incorrect restriction pattern, and stars indicate constructs that consist of dimers (same restriction pattern as monomers). V, vector pX-lacZ.

We chose to make a construct containing a GFP gene with 4 introns and 5 exons (the same construct as described previously [21] but with introns). The introns and exons were defined as separate modules (sequence of the flanking BsaI restriction sites shown in Fig. 2, sequence of the complete modules given in Fig. S1). The 9 fragments were amplified from a cloned GFP gene (for the GFP exons) or from *Arabidopsis thaliana* genomic DNA (for the introns), and cloned into the SmaI site of pUC19spec (a derivative of pUC19 with the ampicillin-resistance β-lactamase gene replaced by a spectinomycin-resistance gene) and sequenced. The recipient expression vector, pX-LacZ (Fig. 2, described previously in [21]) contains two BsaI sites compatible with the first and last exon modules.

To define optimal restriction-ligation conditions, a first experiment was performed using only the nine GFP intron/exon constructs without the acceptor vector. The result of the restriction-ligation is expected to be a 1.17 kb linear fragment containing the assembled GFP exons and introns, in addition to all linear entry vector backbone fragments (2.8 kb). Restriction-ligations were set up by pipetting into a tube 75 ng of each of the 9 plasmids (5 exons, 4 introns), 2.5 units of BsaI enzyme (NEB) and either 2.25 or 15 units of T4 DNA ligase (Promega, 0.75 µl of normal -3 u/µl or high concentration HC ligase - 20 u/µl, respectively) in a total volume of 15 microliters in ligation buffer (Promega). The restriction-ligations were incubated at 37°C for 3 and 6 hours and then run on an agarose gel. The expected 1.17 kb band could be seen only when the ligation was performed with HC ligase, and mostly after a 6 hour ligation (Fig. 2B). To try to improve the amount of assembled ligated product, we modified the restriction-ligation parameters so as to alternate between conditions optimal for annealing of the DNA ends and conditions optimal for enzymatic reactions (digestion or ligation), and for this purpose, performed the restriction-ligation in a thermocycler. Programs were defined with the following steps: incubation for 2 minutes at 37°C and 5 minutes at 16°C, both steps repeated either 25 or 50 times, followed by incubation for 5 minutes at 50°C (final digestion) and then 5 minutes at 80°C (heat inactivation). These conditions were more efficient than a continuous incubation at 37°C because the expected product was visible on a gel even when normal ligase was used, and was highest after 50 cycles

The same conditions as described above were also used with a mix containing the acceptor expression vector in addition to the nine GFP module plasmids (75 ng of each of the 10 plasmids). The ligation was transformed into 100 microliters of chemically competent DH10B cells and 20 µl out of a final volume of 1 ml plated on Kanamycin X-gal plates. For all restriction-ligations performed with BsaI and ligase, the number of white colonies mirrored the efficiency of ligation observed in the ligation assay described above (Table 1). In general, high concentration ligase was more efficient than normal ligase for restriction-ligations performed without cycling, but both normal and high concentration ligases appeared to work well with a program with 50 cycles.

**Table 1:** Assembly of pGFPi from 10 plasmids.

Ligation conditions	neg	3 h 37°C	6 h 37°C		25 cycles		50 cycles	
	blue/white	20 µl plated blue/white	20 µl plated blue/white	estimated total correct	20 µl plated blue/white	estimated total correct	20 µl plated blue/white	estimated total correct
Normal Ligase	1517/0	3/1	1/13	568	1/131	5731	1/379	16581
HC Ligase	985/0	0/72	0/181	7918	1/256	11200	0/211	9231

Restriction-ligation was performed with continuous incubation at 37°C or for 25 or 50 cycles (2 min 37°C+5 min 16°C). 20 µl out of 1 ml were plated. The negative control was performed without BsaI enzyme.

Plasmid DNA was prepared from 12 white colonies for each of 6 transformations (6 hr 37°C, 25 cycles and 50 cycles, each with normal and HC ligase) and was analyzed by gel electrophoresis undigested or digested with XmaI and AvrII. Analysis of undigested DNA (not shown) indicates that 4 out of 72 clones consisted of dimers (vector-insert-vector-insert religated, star in Fig. 2C). Analysis of digested DNA revealed that 67 out of 72 clones had the expected restriction pattern, or 93% of white colonies. When both incorrect inserts and dimers are included, this leads to a success rate of 63 correct colonies out of 72, or 87.5% of all white colonies. By extrapolating this frequency of correct clones to the entire transformation, one can conclude that up to 7918 correct clones were obtained with restriction-ligation performed at 37°C, and up to 16581 correct clones obtained with restriction-ligation performed with cycling.

**Figure 3:**
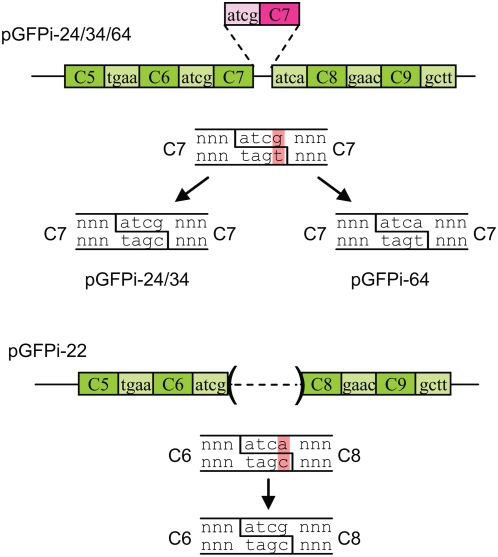
Structure of incorrect GFP constructs and model for their formation. Only the portion between modules 5 and 9 is shown. An additional inserted module (module 7) is shown in pink. Ligation of two DNA ends despite a mismatch in the overhangs leads to a plasmid that can be repaired or segregated in two different sequences. Both alternatives were in fact observed in sequenced plasmids pGFPi-24/34 and 64.

Six clones with a correct restriction digest pattern were sequenced as well as all incorrect constructs. Sequencing confirmed that all 6 clones with the correct restriction pattern had the expected sequence. For the incorrect constructs, 3 clones (24, 34 and 64) contained an insertion of one extra C7 module between modules C7 and C8, while one clone (22) had a deletion of module C7 (Fig. 3). Both types of events can be explained by ligation of inappropriate DNA ends complementary for 3 out of 4 nucleotides.

### DNA shuffling of trypsinogen

**Figure 4:**
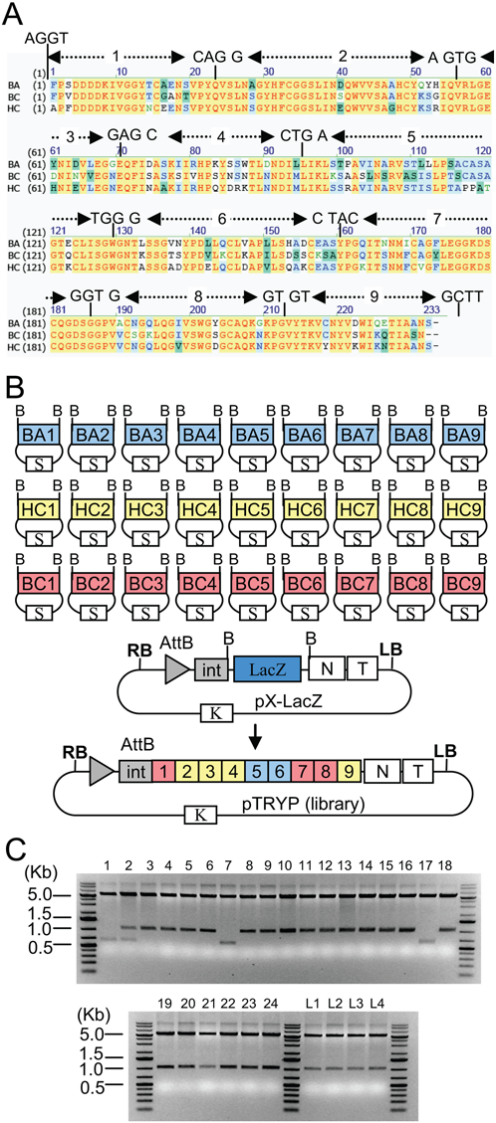
Shuffling of trypsinogen. (A) Alignment of the aminoacid sequence of bovine cationic trypsinogen (BC), bovine anionic trypsinogen (BA) and human cationic trypsinogen (HC). Nucleotide sequence of the chosen recombination sites is shown. (B) Map of the 27 trypsinogen module plasmids, the acceptor vector, and of an example of one of the resulting shuffled construct obtained. B, BsaI restriction site. S, K: spectinomycin and kanamycin resistance genes. RB/LB, T-DNA right and left borders. AttB, Phage C31 recombination site, N tobamoviral 3′ non-translated region, T, Nos terminator. (C) Ethidium bromide-stained gels of 28 minipreps prepared from single colonies (1 to 24) or from 4 libraries (L1–4, approximately 700 clones in each) digested with XmaI (incorrect pattern 1, 2, 7 and 17).

The first set of experiments has allowed to establish restriction-ligation conditions that are efficient enough to allow DNA shuffling. Since we did not have multiple GFP homologues to test the complete shuffling protocol, another protein, trypsinogen, was selected for further experiments. We had earlier tried to express trypsinogen (bovine cationic trypsinogen, UniProtKB database ID P00760) but only low levels of expressed protein were obtained (unpublished results), and hypothesized that low expression might come from either toxicity of trypsinogen to plant tissues or to instability of the protein in plant cells. Therefore, it is possible that related but different trypsinogen proteins might lead to higher level of expression in plants cells. Therefore, the genes for two other related proteins were selected for shuffling: bovine anionic trypsinogen (UniProtKB database ID Q29463) and human cationic trypsinogen (P07477). The coding sequence for bovine cationic trypsinogen was obtained by PCR amplification of exon sequences from calf thymus DNA. The bovine anionic and human cationic trypsinogen genes were chemically synthesized by Entelechon GmbH, with a *Nicotiana* codon usage (sequences given in Fig. S2). The three genes display 66 to 73% identity at the nucleotide level and 74 to 78% identity at the amino acid level. Eight recombination points were chosen on conserved aminoacids (Fig. 4A). These recombination points were selected randomly at positions throughout the genes to define 27 modules (9 sets of 3 modules), with the only requirement that each final module contains a distinct aminoacid sequence. The resulting 27 trypsinogen fragments were amplified by PCR with primers containing flanking BsaI sites, and cloned blunt [24] in the SmaI site of pUC19spec and sequenced.

A restriction-ligation was set up by adding into a single tube 50 ng of each of the 27 trypsinogen fragment constructs (Fig. 4B), 50 ng of vector, 10 units of BsaI enzyme (NEB) and 3 units of T4 DNA ligase (Promega) in a total volume of 15 microliters in ligation buffer (Promega). The restriction-ligation mix was incubated in a thermocycler with the following program: 5 minutes at 37°C and 5 minutes at 16°C, both steps repeated 50 times, followed by incubation for 5 minutes at 50°C and 5 minutes at 80°C (trypsin shuffling experiment 1, ts1, Table 2). The ligation was transformed in 100 µl chemically competent cells and 50 µl out of a final volume of 1 ml plated on Kanamycin X-gal plates. After counting the number of white colonies per plate and extrapolating to the whole transformation, a total of 7320 white colonies were obtained. Plasmid DNA from 24 white colonies was analyzed by gel electrophoresis (of cut and uncut DNA); four clones had an incorrect restriction pattern (Fig. 4C) and two were dimers (not shown). The 18 correct clones were sequenced and found to have correctly assembled inserts and all of these were different (structure of all sequenced clones shown in Fig. S3). This shows that out of the 7320 colonies, 5490 colonies are estimated to contain correct constructs.

**Table 2:** Trypsinogen shuffling.

Exp n°	modules	program	blue	white	minipreps with correct restriction pattern	dimers	Correct clones, sequenced	Incorrect clones, sequenced
ts1	mod1	37°C 5 min 16°C 5 min, 50×	10	7320	20/24	2/24	18/18	4/4
ts2	mod1	37°C 5 min 16°C 5 min, 50×	59	10190	21/24	1/24	20/20	3/3
ts3	mod1	37°C 5 min 20°C 5 min, 50×	nd	nd	22/24	1/24	none	2/2
ts4	mod1	37°C 5 min 25°C 5 min, 50×	nd	nd	22/24	1/24	none	2/2
ts5	mod1	37°C 5 min 16°C 5 min, 50×	104	10914	45/48	1/48	none	3/3
ts6	mod1	37°C 5 min 16°C 5 min, 50×	112	13712	45/48	0/48	none	3/3
ts30	mod1	37°C 6 hr, hc	0	11730	21/24	1/24	none	3/3
ts15	mod2	37°C 2 min 16°C 5 min, 50×	35	12432	23/24	0/24	23/23	1/1
ts16	mod2	37°C 5 min 16°C 5 min, 50×	33	14249	21/24	1/24	20/20	3/3
ts23	mod2	37°C 2 min 20°C 5 min, 50×	40	6063	24/24	0/24	none	0/0
ts24	mod2	37°C 2 min 25°C 5 min, 50×	0	4809	24/24	3/24	none	0/0
ts25	mod2	37°C 2 min 30°C 5 min, 50×	8	6353	23/24	2/24	none	1/1
ts26	mod2	37°C 2 min 16°C 5 min, 50×	16	6538	24/24	3/24	none	0/0
ts27	mod2	37°C 6 hr	0	8835	23/24	1/24	none	1/1
ts28	mod2	37°C 6 hr, hc	0	8095	24/24	0/24	none	0/0
ts29	mod2	37°C 2 min 30°C 5 min, hc, 50×	10	9425	23/24	2/24	none	1/1

Constructs were made from a first set of trypsinogen modules (mod1, the junction between modules 2 to 3 is agtg) or a second set of modules (mod2, junction between modules 2 to 3 is agtc). White and blue are the total number of colonies obtained per transformation (extrapolated from the number of colonies obtained per plate). All restriction-ligations were performed using equimolar amount of insert and vector except for ts1 that was made using three times less vector (50 ng) than insert. Dimers were identified by running uncut DNA on an agarose gel. Programs used either 6 hours at 37°C (37°C 6 hr) or 50 cycles (conditions given in program); all programs are followed by digestion 5 min at 50°C and heat inactivation 5 min at 80°C. hc, use of high concentration ligase.

Shuffling was repeated using the same amount of inserts but three times more vector (to be in the same molar ratio as the inserts (shuffling experiment ts2, Table 2), and 10190 white colonies were obtained, with an estimated number or 8492 correct constructs. In order to get a complete library (the maximal theoretical diversity for shuffling 3 genes in nine fragments is 19,683) one only needs to transform three separate 15 µl reactions or use more efficient competent cells (for example electrocompetent).

### Optimization of ligation parameters and module design

**Figure 5:**
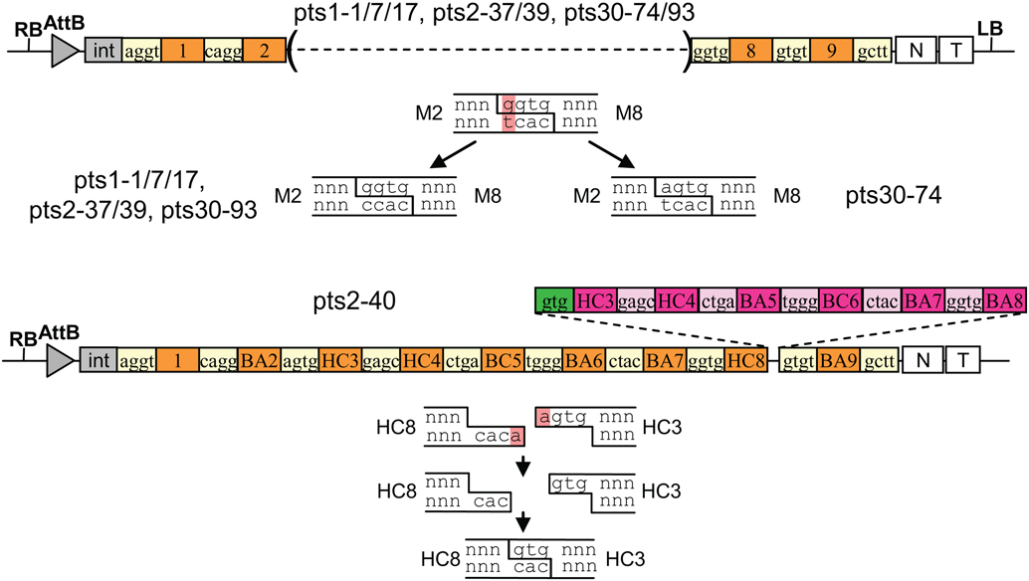
Structure of incorrect trypsinogen constructs and models for their formation. Additional inserted modules in clone ts2–40 are shown in pink, and a religated overhang with 3 nucleotides shown in green. For this clone, exonuclease removal of an A resulted in two complementary 3-nucleotide overhangs that could be ligated.

All plasmids with incorrect restriction pattern were sequenced. The majority of incorrect clones (clones ts1–1/7/17, ts2–37 and 39) had a deletion of 5 modules (modules 3 to 7, Fig. 5). As for the incorrect GFP constructs, these can be explained by ligation of DNA ends complementary for 3 out of the 4 nucleotides, in this case between modules 3 (sequence of the top strand: agtg) and 8 (ggtg). Finally, one trypsinogen construct contained 6 extra modules (modules 3 to 8) between modules 8 and 9. In this case, exonucleolytic removal of one terminal base from the 5′ end of each DNA overhang led to two complementary three base extensions that were able to anneal and become ligated (Fig. 5). This base removal can be explained by the presence of trace amount of a contaminating exonuclease in one of the components introduced in the ligation mix (the plasmids, the enzymes or the buffer).

Two approaches were used to try to further improve the efficiency of cloning. One consisted of modifying the ligation conditions, in particular the temperature, so as to minimize ligation of ends that are not perfectly complementary. For example, shuffling was performed using programs in which the 16°C incubation was increased from 16 to 20, 25, 30 or 37°C (experiments ts3 to ts6 and ts30, Table 2). However, these modifications did not significantly affect cloning efficiency.

The second approach consisted of modifying the sequence joining modules 2 to and 3 (which is involved in inappropriate ligation to module 8): the sequence was changed from agtg to agtc (a silent substitution) to prevent inappropriate ligation to module 8. This means that six modules BA2, BC2, HC2, BA3, BC3 and HC3, had to be recloned. Shuffling was then repeated with the 6 new modules using a range of different restriction-ligation conditions (experiments ts15, ts16, ts23 to 29, Table 2). This modification led to an increase in clones with the correct restriction pattern from 91% to 97%. After substracting the amount of clones that contained dimers, the overall number of correct clones increased from 87.5% to 91.5%. Constructs with the incorrect pattern obtained with the new modules were also sequenced. The majority of incorrect clones were generated as a result of exonucleolytic removal of at least one base at the 3′ end of the overhang (Fig. S4).

All clones with a correct restriction pattern from cloning experiments ts15 and 16 (43 clones) were sequenced. All constructs were found to contain shuffled trypsinogen genes as expected, and all were different (structure in Fig. S3). None contained any single nucleotide mutation. This is expected since these constructs are assembled without using PCR amplification.

### Screening of the shuffled trypsinogen constructs

**Figure 6:**
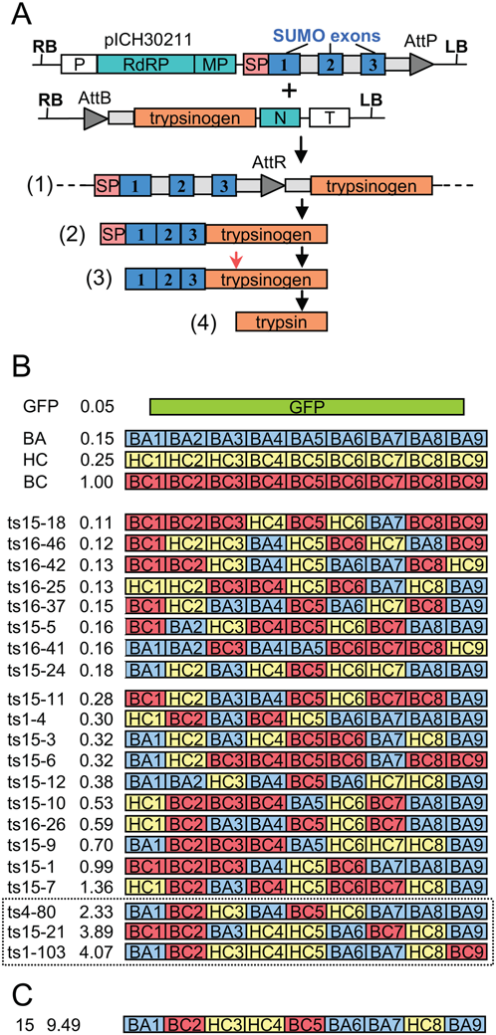
Activity assay of the shuffled trypsinogen constructs. (A) Three constructs (in *Agrobacterium*) are coinfiltrated for each trypsinogen construct: the 5′ viral vector module (pICH30211), a trypsinogen construct, and an integrase construct (not shown). *In planta* recombination leads to formation of an assembled construct (1) which leads to viral expression of a fusion protein (2) containing a signal peptide (SP), *Arabidopsis thaliana* SUMO exons, and trypsinogen. The signal peptide is cleaved upon import through the ER (3), and trypsin is obtained by autocatalytic cleavage of the proprotein (red arrow). Grey boxes represent introns. (B) Activity and structure of some of the constructs obtained from the first round of shuffling (name, column 1 and activity, column 2), activity expressed relative to activity of bovine cationic trypsinogen (BC). Activity for the parents (BA, HC, BC) was also measured (from corresponding constructs infiltrated as a control). GFP is used as a negative control. The last 3 constructs (boxed) were used for a second round of shuffling. (C) Best construct obtained with the second round of shuffling.

The 81 sequenced constructs (all different) and 87 constructs analyzed by restriction digest but not sequenced were transformed in *Agrobacterium* strain GV3101:pMP90. In addition, a library of unscreened recombinant plasmids was directly transformed in *Agrobacterium*, and 53 *Agrobacterium* colonies were picked and grown separately for infiltration. In addition, two other *Agrobacterium* strains were grown: a strain containing a 5′ viral vector containing an Arabidopsis SUMO gene and a strain containing a construct for plant expression of recombinase [25]. The outcome of coinfiltration of three strains in plant tissues (the 5′ vector, the recombinase, and a trypsinogen construct 3′ vector) leads to recombination in plant tissues of the 5′ construct and the trypsinogen construct, and to expression and secretion in the apoplast of a fusion protein: Arabidopsis SUMO-shuffled trypsinogen (Fig. 6A). Autocatalytic conversion of trypsinogen to trypsin then occurs (either in plant tissues or during extraction).

The 221 different shuffled trypsinogen constructs were infiltrated. Three constructs containing the parental genes were also infiltrated. Plant tissue was harvested at 7 days post infiltration (dpi). Trypsin enzymatic activity was determined using a colorimetric assay based on the conversion of a colorless substrate, BAPNA, into a yellow product by digestion with trypsin. Four clones (two of the previously sequenced constructs, clones ts15–7 and ts15–21, one from the characterized but non-sequenced miniprep, clone ts4–80, and one from the library of non-characterized plasmids, clone ts1–103) were found to provide a higher level of activity than the bovine trypsinogen construct control, with the best clone 103, displaying approximately 4 fold higher activity (Fig. 6B). Both non-sequenced clones were then sequenced (structure shown in Fig. 6B).

A second round of shuffling was performed using information from the 3 best clones obtained (ts15–21, ts4–80 and ts1–103). Shuffling was performed by setting up a restriction-ligation containing modules in the same molar ratio as in the three selected parents combined (module set 1: BA1/BC1/HC1, 100/50/0 ng; module set 2: BA2/BC2/HC2: 0/150/0 ng; etc). Since not all of the 27 modules are used, the number of theoretical possible combinations is only 256 different constructs. Nevertheless, one construct with nine fold higher activity than bovine trypsinogen was obtained after screening 24 new recombinants (Fig. 6C). Preliminary data (not shown) suggests that the high activity of these clones is due to an increase of specific activity toward the BAPNA substrate rather than an increase in the amount of expressed protein; more precise quantification will be the subject of a separate study.

## Discussion

We have shown here that inserts from nine separate plasmids (or nine sets of modules) can be easily and efficiently assembled and cloned in an acceptor vector in one step and one tube. The efficiency of this protocol comes from the fact that the only stable product(s) issued from the restriction-ligation are the desired product(s) [21]; these products are formed continuously with each cycle and with increasing length of incubation. Assembly was shown to be efficient with two independent sets of BsaI restriction sites overhangs, one set with the GFP construct, and the second set with trypsinogen. Sequencing of the constructs with incorrect restriction pattern obtained with both sets has allowed to draw some conclusions as to how these overlaps should be selected to maximize cloning efficiency. The majority of incorrect constructs for both experiments were found to occur as a result of ligation of two DNA ends complementary for three consecutive out of the four nucleotides of the overhang. This occurrence can be explained by inappropriate ligation of improperly annealed ends. An alternative explanation would consist of removal of a terminal nucleotide from one of the DNA ends by a contaminating exonuclease, and ligation of only one of the DNA strands of the annealed product. Whatever the mechanism, this occurrence can be reduced by selecting a set of recombination sites in which none of the site shares three consecutive nucleotides with any other selected site. This is usually not a problem since a large number of possible sequences, 240 (256 theoretically possible sequences, minus the 16 palindromes that should be excluded), can be chosen from. For each additional site to select, the sequence of previously selected sites or their complement should be excluded as well (use of 2 sites with complementary sequences would allow one fragment to be inappropriately ligated at the wrong position and in the opposite orientation). With a set of overlaps chosen according to these criteria (the trypsinogen second set of modules), 97% of constructs obtained contained only correctly ligated DNA ends. This efficiency suggests that it is likely that more than nine fragments could be ligated together and still result in a high percentage of correct constructs.

Sequencing of 87 constructs with a correct restriction pattern (81 trypsinogen constructs and 6 GFP constructs) showed that none contained any single mutation in the shuffled genes. This is expected because shuffling is performed without the use of PCR; the modules in the intermediate constructs are made using PCR but are then sequenced before being used for restriction-ligation assembly.

A second reason for the efficiency of the assembly protocol is that the number of procedures performed on DNA has been brought down to a minimum. Indeed, any manipulation performed on DNA, including extraction, digestion, buffer exchange, dephosphorylation, DNA precipitation, column purification, or any other DNA manipulation procedure, is likely to result in some amount of DNA damage and to loss of some of the DNA. With the protocol described here, the plasmids used for assembly are not pre-digested but simply added to the restriction-ligation mix. Only one step and one buffer are used, and the time between digestion and ligation is brought to a minimum. No purification step is required between DNA preparation of the input modules and transformation of the library in competent cells. Using undigested plasmids for this procedure rather than digested gel-purified DNA fragments has an added advantage: it allows estimating the relative DNA concentration of the modules (which might vary in size significantly) more precisely; this is because the relative size difference between modules is much lower for plasmids than for purified inserts. This precision if very important when it comes to ligating many fragments since a module present in too low or too high amount would become a limiting factor and reduce the number of final clones. Unlike for standard cloning, where only one clone is usually required, obtaining the maximum number of independent recombinant plasmids is a necessity for DNA shuffling.

The protocol described here has two applications: (1) making constructs and (2) DNA shuffling. Regarding the first application, the ability to assemble in one step a construct from 10 different plasmids should allow much more flexibility and efficiency in making constructs than is now possible. Cloning strategies that require many successive steps can now by done in two steps: one being preparation of the intermediate constructs and the second, assembly of the final construct. The cloning protocol described here is in fact an extension of the ‘Golden Gate’ cloning protocol described earlier [21]. A protocol based on ligation-independent cloning has been reported that also allows cloning nine fragments into a vector [26], but efficiency was lower at about 17%. Moreover, the protocol is based on assembly of PCR products rather than of sequenced inserts in plasmids, which means that a portion of the constructs obtained will contain mutations derived from the primers or the PCR amplification. A protocol has also been reported for the cloning of four fragments using a restriction-ligation using type II enzymes that produce compatible ends, such as EcoRI and MfeI [27]. However, this strategy required adding eight different enzymes to the restriction-ligation mix for ligation of just four fragments. Ligation of nine fragments in one vector would require the simultaneous use of 20 different enzymes in the same mix. Finding such a combination would impose extreme limitations on the design of any cloning experiment.

The application of this cloning protocol to DNA shuffling results in a protocol that we call ‘Golden Gate shuffling’. The use of Type IIs enzymes for DNA shuffling has been reported before [17], [18], [19], [20], [28]. However, assembly of the modules was performed from gel purified pre-digested DNA fragments. As a result, in many cases, ligation had to be done module set by module set in consecutive steps, which led to a low amount of assembled product, often requiring PCR amplification of the library before cloning in the expression vector. In contrast, with Golden gate shuffling, once modules are made, assembling a defined set of modules is easy to perform. A first round of shuffling might provide a number of improved constructs that an experimenter might want to subject to a second round of shuffling. In that case, performing the second round of shuffling may consist or performing a one-tube restriction-ligation with different relative ratios of already made input modules. Another advantage comes from the fact that PCR is not required for assembly of the final library; this is useful since no PCR mutations will be present in the final library. Because of this feature, theoretically, large genes can therefore be shuffled. Another advantage of this technology is that shuffling can be done between parental templates that have no homology at all, one application being exon shuffling (as previously described by [28]). The only requirement is the presence of 4 nt at the chosen junction points. This means only one fixed aminoacid at each junction point.

Shuffling of three genes divided in 9 modules (9 sets of modules, each set containing 3 modules) provides a theoretical number of variants of 19,683 and shuffling of four or five genes would provide a maximal theoretical diversity of 262,144 and 2 million combinations, respectively. However, Golden Gate shuffling does not need to be limited to assembly of pre-made sequenced modules. In fact, it can be used to combine together modules sets that have been prepared with different shuffling protocols. For example, one set of modules might be made using any of the existing DNA shuffling protocols and might consist of thousands or even millions of variants. These module sets can be combined together with other less variable module sets, depending on the need of the experimenter. At the same time, not all sets of modules need to contain the same amount of modules. For example, one module set might consist of only one module of defined sequence that is used as a linker between two highly variable sets of modules.

Therefore, the flexibility and efficiency of Golden Gate shuffling as well as its compatibility and complementarity with other DNA shuffling protocols should make it a valuable tool for molecular evolution.

## Materials and Methods

### Molecular biology techniques

Chemically competent cells were prepared as described earlier [21]. Agrobacterium infiltration of plant tissue has been described in [25]. Plasmid DNA minipreps were made using the Nucleospin Plasmid Quick Pure kit from Macherey-Nagel, Düren, Germany.

### Trypsin enzymatic assay

100 mg of plant tissue was ground in liquid nitrogen and mixed with 300 ml of extraction buffer (0.15 M Tris pH 8.0, 2 mM EDTA). The extract was incubated for 10 minutes on ice and centrifuged for 15 minutes at 13,000 rpm. 20 µl of the supernatant was mixed with 20 µl of 2 mM BAPNA substrate (Sigma Aldrich). OD was read every 5 min from 5 to 45 min using a BioTek ELx808 Absorbance Microplate Reader with a 405 nm filter. Enzymatic activity was measured in the linear part of the curve (5–20 min) as the rate of the curve, to which background activity of uninfiltrated WT tissue was substracted. Activity was then expressed relative to activity of the bovine cationic trypsinogen parent construct.

## Supporting Information

Figure S1 Sequence of GFP intron and exon modules and of the final assembled construct. The sequence of the 5 GFP exon modules, the 4 intron modules and of the final assembled GFP construct is given.
(0.04 MB PPT)

Figure S2 Alignment of the nucleotide sequences of bovine cationic trypsinogen, bovine anionic trypsinogen, and human cationic trypsinogen. The nucleotide sequence alignment of bovine cationic trypsinogen (BC), bovine anionic trypsinogen (BA), and human cationic trypsinogen (HC) is shown. Sequences selected as recombination sites/cloning sites are boxed.
(0.24 MB PPT)

Figure S3 Structure of correct sequenced trypsinogen constructs. Constructs with a correct restriction pattern from shuffling experiments ts1, ts2, ts15 and ts16 were sequenced. Their sequence is given as well as the sequence of two other clones with high activity, ts4–80 and 1s1–103.
(0.22 MB PPT)

Figure S4 Structure of incorrect trypsinogen constructs obtained with the second set of modules. This figure shows the structure of incorrect trypsinogen constructs obtained with the second set of modules (mod2), and proposes models to explain their formation.
(0.11 MB PPT)

## References

[1] Cadwell RC, Joyce GF (1992). Randomization of genes by PCR mutagenesis.. PCR Methods and Applications.

[2] Wang TW, Zhu H, Ma XY, Zhang T, Ma YS (2006). Mutant library construction in directed molecular evolution: casting a wider net.. Molecular Biotechnology.

[3] Sen S, Venkata Dasu V, Mandal B (2007). Developments in directed evolution for improving enzyme functions.. Applied biochemistry and biotechnology.

[4] Lutz S, Patrick WM (2004). Novel methods for directed evolution of enzymes: quality, not quantity.. Current Opinion in Biotechnology.

[5] Stemmer WPC (1994). DNA shuffling by random fragmentation and reassembly: in vitro recombination for molecular evolution.. Proc Natl Acad Sci U S A.

[6] Stemmer WP (1994). Rapid evolution of a protein in vitro by DNA shuffling.. Nature.

[7] Crameri A, Raillard SA, Bermudez E, Stemmer WP (1998). DNA shuffling of a family of genes from diverse species accelerates directed evolution.. Nature.

[8] Coco WM, Levinson WE, Crist MJ, Hektor HJ, Darzins A (2001). DNA shuffling method for generating highly recombined genes and evolved enzymes.. Nature Biotechnology.

[9] Ness JE, Kim S, Gottman A, Pak R, Krebber A (2002). Synthetic shuffling expands functional protein diversity by allowing amino acids to recombine independently.. Nature Biotechnology.

[10] Coco WM, Encell LP, Levinson WE, Crist MJ, Loomis AK (2002). Growth factor engineering by degenerate homoduplex gene family recombination.. Nature Biotechnology.

[11] Zhao H, Giver L, Shao Z, Affholter JA, Arnold FH (1998). Molecular evolution by staggered extension process (StEP) in vitro recombination.. Nature Biotechnology.

[12] Eggert T, Funke SA, Rao NM, Acharya P, Krumm H (2005). Multiplex-PCR-based recombination as a novel high-fidelity method for directed evolution.. Chembiochem.

[13] Jézéquel L, Loeper J, Pompon D (2008). Sequence-independent construction of ordered combinatorial libraries with predefined crossover points.. Biotechniques.

[14] Ostermeier M, Shim JH, Benkovic SJ (1999). A combinatorial approach to hybrid enzymes independent of DNA homology.. Nature Biotechnology.

[15] Volkov AA, Shao Z, Arnold FH (1999). Recombination and chimeragenesis by in vitro heteroduplex formation and in vivo repair.. Nucleic acids research.

[16] Sieber V, Martinez CA, Arnold FH (2001). Libraries of hybrid proteins from distantly related sequences.. Nature Biotechnology.

[17] Hiraga K, Arnold FH (2003). General method for sequence-independent site-directed chimeragenesis.. Journal of molecular biology.

[18] Meyer MM, Hiraga K, Arnold FH (2006). Combinatorial recombination of gene fragments to construct a library of chimeras.. Current protocols in protein science.

[19] Richardson TH, Tan X, Frey G, Callen W, Cabell M (2002). A novel, high performance enzyme for starch liquefaction. Discovery and optimization of a low pH, thermostable alpha-amylase.. The Journal of Biological Chemistry.

[20] Trefzer A, Jungmann V, Molnár I, Botejue A, Buckel D (2007). Biocatalytic conversion of avermectin to 4′-oxo-avermectin: improvement of cytochrome p450 monooxygenase specificity by directed evolution.. Applied and Environmental Microbiology.

[21] Engler C, Kandzia R, Marillonnet S (2008). A one pot, one step, precision cloning method with high throughput capability.. PLoS ONE.

[22] Kotera I, Nagai T (2008). A high-throughput and single-tube recombination of crude PCR products using a DNA polymerase inhibitor and type IIS restriction enzyme.. J Biotechnol.

[23] Woodard SL, Mayor JM, Bailey MR, Barker DK, Love RT (2003). Maize (Zea mays)-derived bovine trypsin: characterization of the first large-scale, commercial protein product from transgenic plants.. Biotechnol Appl Biochem.

[24] Bolchi A, Ottonello S, Petrucco S (2005). A general one-step method for the cloning of PCR products.. Biotechnol Appl Biochem.

[25] Marillonnet S, Giritch A, Gils M, Kandzia R, Klimyuk V (2004). In planta engineering of viral RNA replicons: efficient assembly by recombination of DNA modules delivered by Agrobacterium.. Proc Natl Acad Sci U S A.

[26] Li MZ, Elledge SJ (2007). Harnessing homologous recombination in vitro to generate recombinant DNA via SLIC.. Nature Methods.

[27] Cost G (2007). Enzymatic ligation assisted by nucleases: simultaneous ligation and digestion promote the ordered assembly of DNA.. Nature Protocols.

[28] Silverman J, Liu Q, Bakker A, To W, Duguay A (2005). Multivalent avimer proteins evolved by exon shuffling of a family of human receptor domains.. Nature Biotechnology.

