# Supplementary material for: Golden Gate Shuffling: A One-Pot DNA Shuffling Method Based on Type IIs Restriction Enzymes
Source: PLoS One. 2009 May 14;4(5):e5553. doi: 10.1371/journal.pone.0005553 (PMC2677662; doi:10.1371/journal.pone.0005553)
Supplement: Figure S1 — Sequence of GFP intron and exon modules and of the final assembled construct. The sequence of the 5 GFP exon modules, the 4 intron modules and of the final assembled GFP construct is given. (0.04 MB PPT) [file pone.0005553.s001.ppt]

## Slide 1
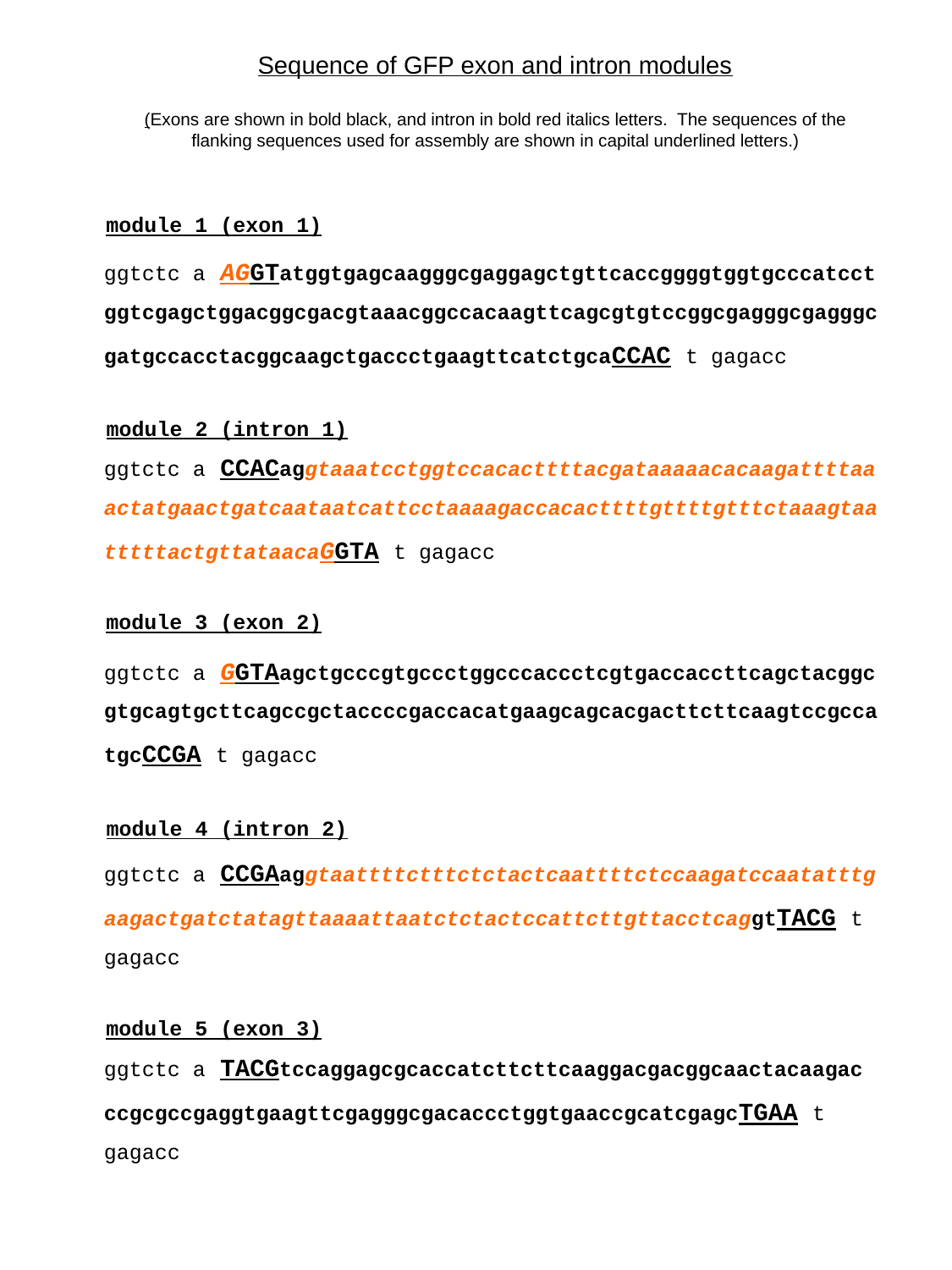

Sequence of GFP exon and intron modules
(Exons are shown in bold black, and intron in bold red italics letters. The sequences of the flanking sequences used for assembly are shown in capital underlined letters.)
module 1 (exon 1)
ggtctc a AGGTatggtgagcaagggcgaggagctgttcaccggggtggtgcccatcct
ggtcgagctggacggcgacgtaaacggccacaagttcagcgtgtccggcgagggcgagggcgatgccacctacggcaagctgaccctgaagttcatctgcaCCAC t gagacc
module 2 (intron 1)
ggtctc a CCACaggtaaatcctggtccacacttttacgataaaaacacaagattttaa
actatgaactgatcaataatcattcctaaaagaccacacttttgttttgtttctaaagtaatttttactgttataacaGGTA t gagacc
module 3 (exon 2)
ggtctc a GGTAagctgcccgtgccctggcccaccctcgtgaccaccttcagctacggc
gtgcagtgcttcagccgctaccccgaccacatgaagcagcacgacttcttcaagtccgccatgcCCGA t gagacc
module 4 (intron 2)
ggtctc a CCGAaggtaattttctttctctactcaattttctccaagatccaatatttg
aagactgatctatagttaaaattaatctctactccattcttgttacctcaggtTACG t gagacc
module 5 (exon 3)
ggtctc a TACGtccaggagcgcaccatcttcttcaaggacgacggcaactacaagac
ccgcgccgaggtgaagttcgagggcgacaccctggtgaaccgcatcgagcTGAA t gagacc

## Slide 2
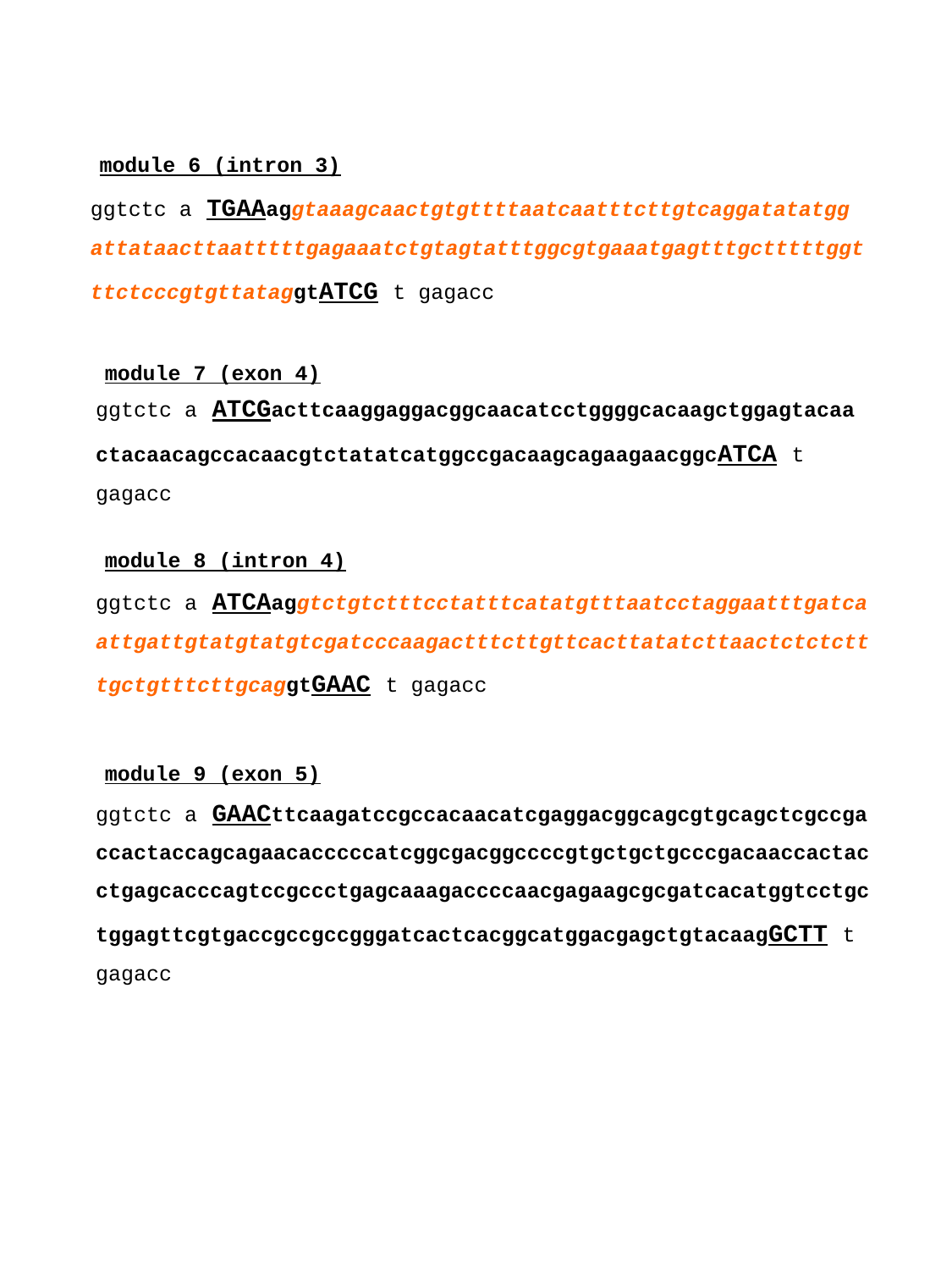

module 6 (intron 3)
ggtctc a TGAAaggtaaagcaactgtgttttaatcaatttcttgtcaggatatatgg
attataacttaatttttgagaaatctgtagtatttggcgtgaaatgagtttgctttttggtttctcccgtgttataggtATCG t gagacc
module 7 (exon 4)
ggtctc a ATCGacttcaaggaggacggcaacatcctggggcacaagctggagtacaa
ctacaacagccacaacgtctatatcatggccgacaagcagaagaacggcATCA t gagacc
module 8 (intron 4)
ggtctc a ATCAaggtctgtctttcctatttcatatgtttaatcctaggaatttgatca
attgattgtatgtatgtcgatcccaagactttcttgttcacttatatcttaactctctctttgctgtttcttgcaggtGAAC t gagacc
module 9 (exon 5)
ggtctc a GAACttcaagatccgccacaacatcgaggacggcagcgtgcagctcgccga
ccactaccagcagaacacccccatcggcgacggccccgtgctgctgcccgacaaccactacctgagcacccagtccgccctgagcaaagaccccaacgagaagcgcgatcacatggtcctgctggagttcgtgaccgccgccgggatcactcacggcatggacgagctgtacaagGCTT t gagacc

## Slide 3
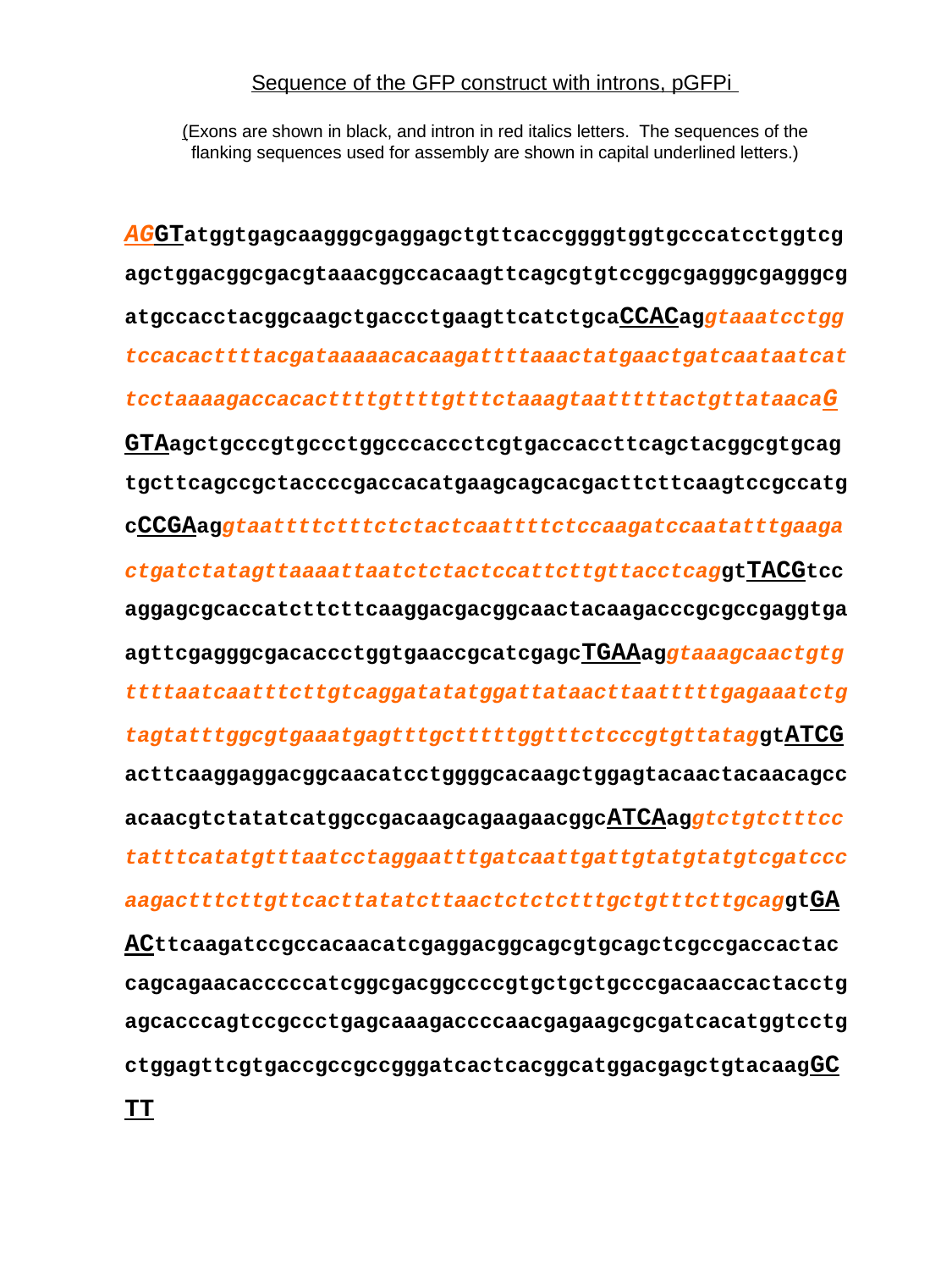

Sequence of the GFP construct with introns, pGFPi
(Exons are shown in black, and intron in red italics letters. The sequences of the flanking sequences used for assembly are shown in capital underlined letters.)
AGGTatggtgagcaagggcgaggagctgttcaccggggtggtgcccatcctggtcgagctggacggcgacgtaaacggccacaagttcagcgtgtccggcgagggcgagggcgatgccacctacggcaagctgaccctgaagttcatctgcaCCACaggtaaatcctggtccacacttttacgataaaaacacaagattttaaactatgaactgatcaataatcattcctaaaagaccacacttttgttttgtttctaaagtaatttttactgttataacaGGTAagctgcccgtgccctggcccaccctcgtgaccaccttcagctacggcgtgcagtgcttcagccgctaccccgaccacatgaagcagcacgacttcttcaagtccgccatgcCCGAaggtaattttctttctctactcaattttctccaagatccaatatttgaagactgatctatagttaaaattaatctctactccattcttgttacctcaggtTACGtccaggagcgcaccatcttcttcaaggacgacggcaactacaagacccgcgccgaggtgaagttcgagggcgacaccctggtgaaccgcatcgagcTGAAaggtaaagcaactgtgttttaatcaatttcttgtcaggatatatggattataacttaatttttgagaaatctgtagtatttggcgtgaaatgagtttgctttttggtttctcccgtgttataggtATCGacttcaaggaggacggcaacatcctggggcacaagctggagtacaactacaacagccacaacgtctatatcatggccgacaagcagaagaacggcATCAaggtctgtctttcctatttcatatgtttaatcctaggaatttgatcaattgattgtatgtatgtcgatcccaagactttcttgttcacttatatcttaactctctctttgctgtttcttgcaggtGAACttcaagatccgccacaacatcgaggacggcagcgtgcagctcgccgaccactaccagcagaacacccccatcggcgacggccccgtgctgctgcccgacaaccactacctgagcacccagtccgccctgagcaaagaccccaacgagaagcgcgatcacatggtcctgctggagttcgtgaccgccgccgggatcactcacggcatggacgagctgtacaagGCTT
