# Supplementary material for: Golden Gate Shuffling: A One-Pot DNA Shuffling Method Based on Type IIs Restriction Enzymes
Source: PLoS One. 2009 May 14;4(5):e5553. doi: 10.1371/journal.pone.0005553 (PMC2677662; doi:10.1371/journal.pone.0005553)
Supplement: Figure S2 — Alignment of the nucleotide sequences of bovine cationic trypsinogen, bovine anionic trypsinogen, and human cationic trypsinogen. The nucleotide sequence alignment of bovine cationic trypsinogen (BC), bovine anionic trypsinogen (BA), and human cationic trypsinogen (HC) is shown. Sequences selected as recombination sites/cloning sites are boxed. (0.24 MB PPT) [file pone.0005553.s002.ppt]

## Slide 1
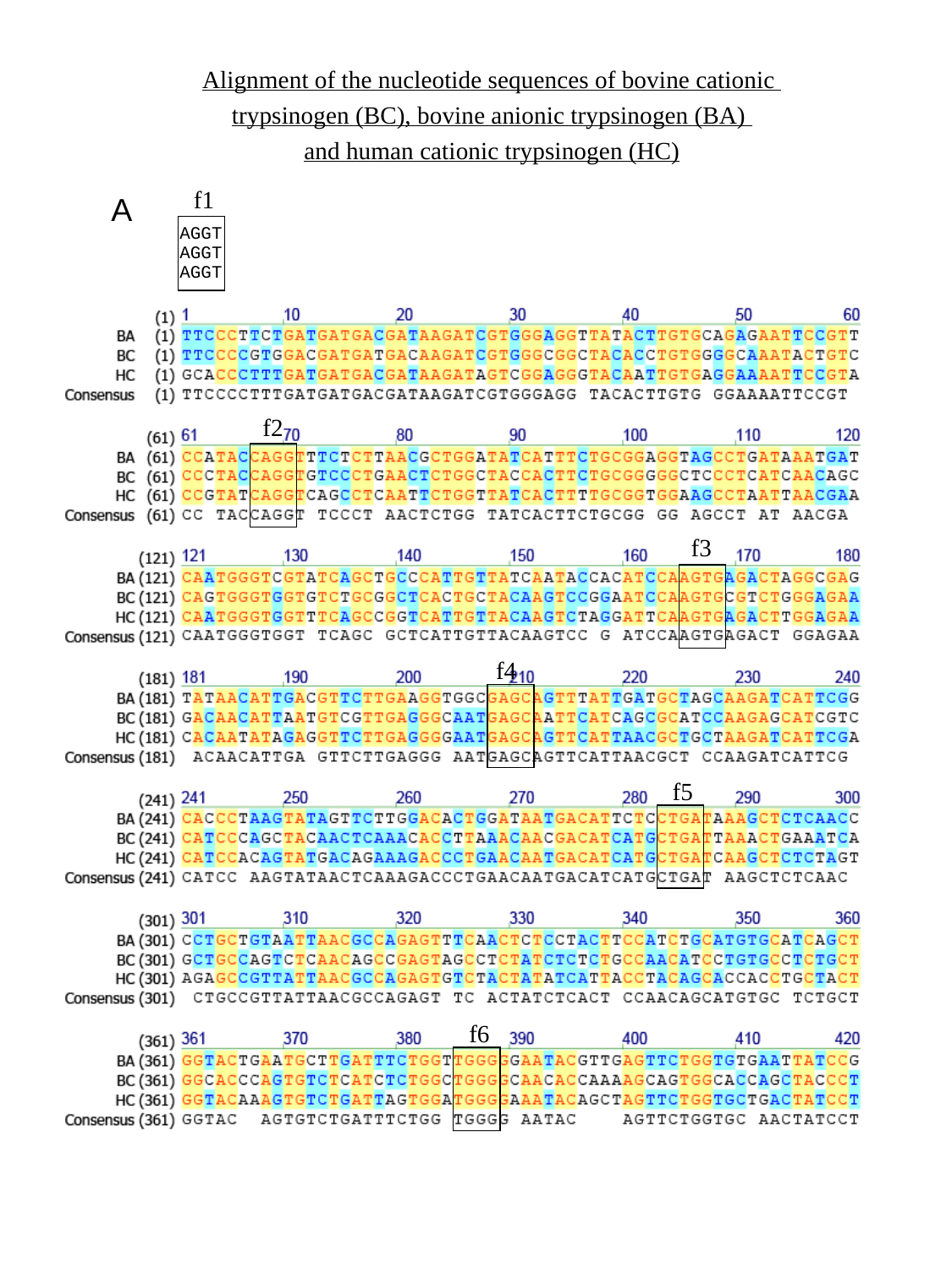

Alignment of the nucleotide sequences of bovine cationic
trypsinogen (BC), bovine anionic trypsinogen (BA)
and human cationic trypsinogen (HC)
f1
A
AGGT
AGGT
AGGT
f2
f3
f4
f5
f6

## Slide 2
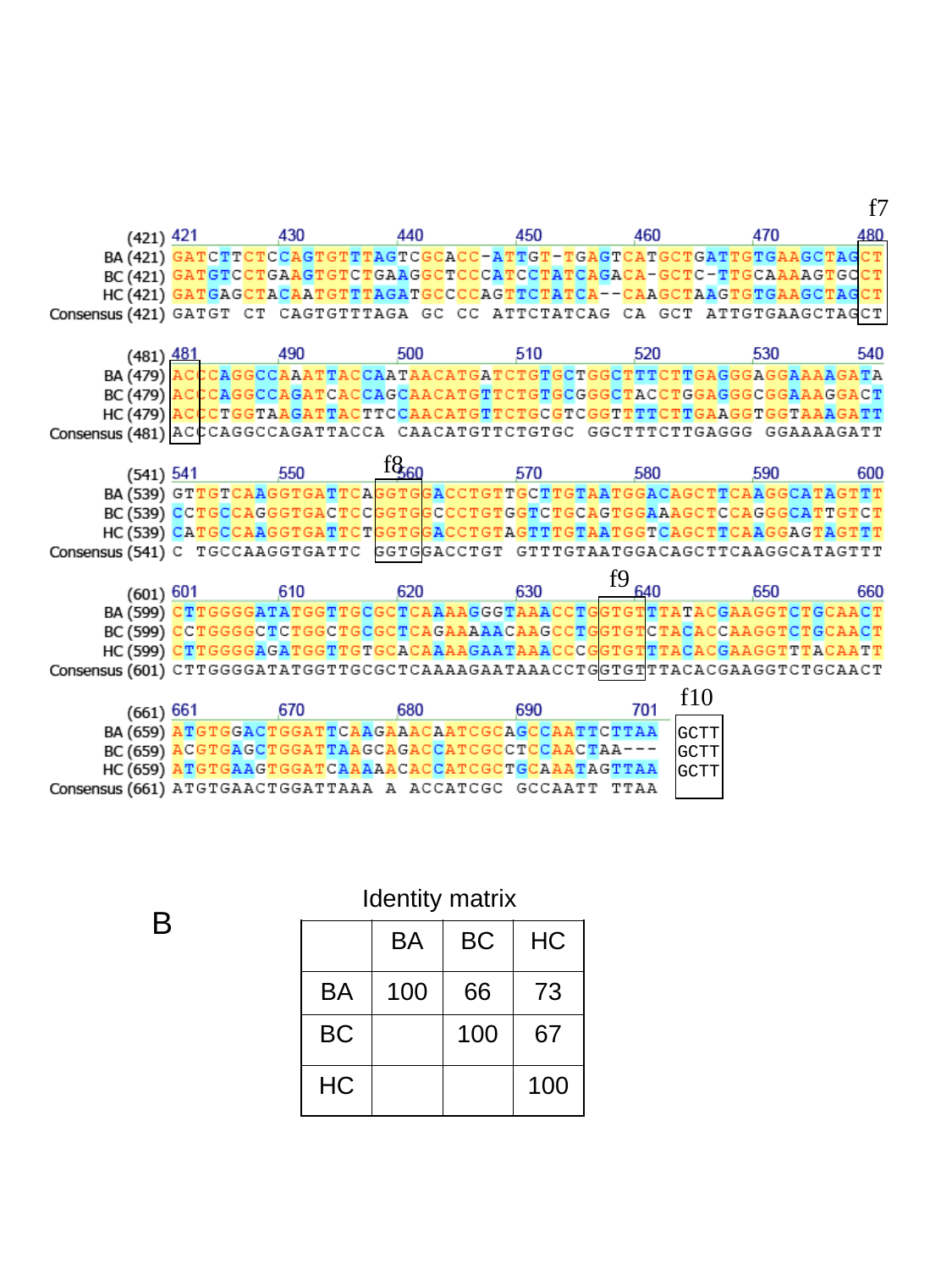

f7
f8
f9
f10
GCTT
GCTT
GCTT
Identity matrix
B
| | BA | BC | HC |
| --- | --- | --- | --- |
| BA | 100 | 66 | 73 |
| BC | | 100 | 67 |
| HC | | | 100 |
