# Supplementary material for: Golden Gate Shuffling: A One-Pot DNA Shuffling Method Based on Type IIs Restriction Enzymes
Source: PLoS One. 2009 May 14;4(5):e5553. doi: 10.1371/journal.pone.0005553 (PMC2677662; doi:10.1371/journal.pone.0005553)
Supplement: Figure S3 — Structure of correct sequenced trypsinogen constructs. Constructs with a correct restriction pattern from shuffling experiments ts1, ts2, ts15 and ts16 were sequenced. Their sequence is given as well as the sequence of two other clones with high activity, ts4–80 and 1s1–103. (0.22 MB PPT) [file pone.0005553.s003.ppt]

## Slide 1
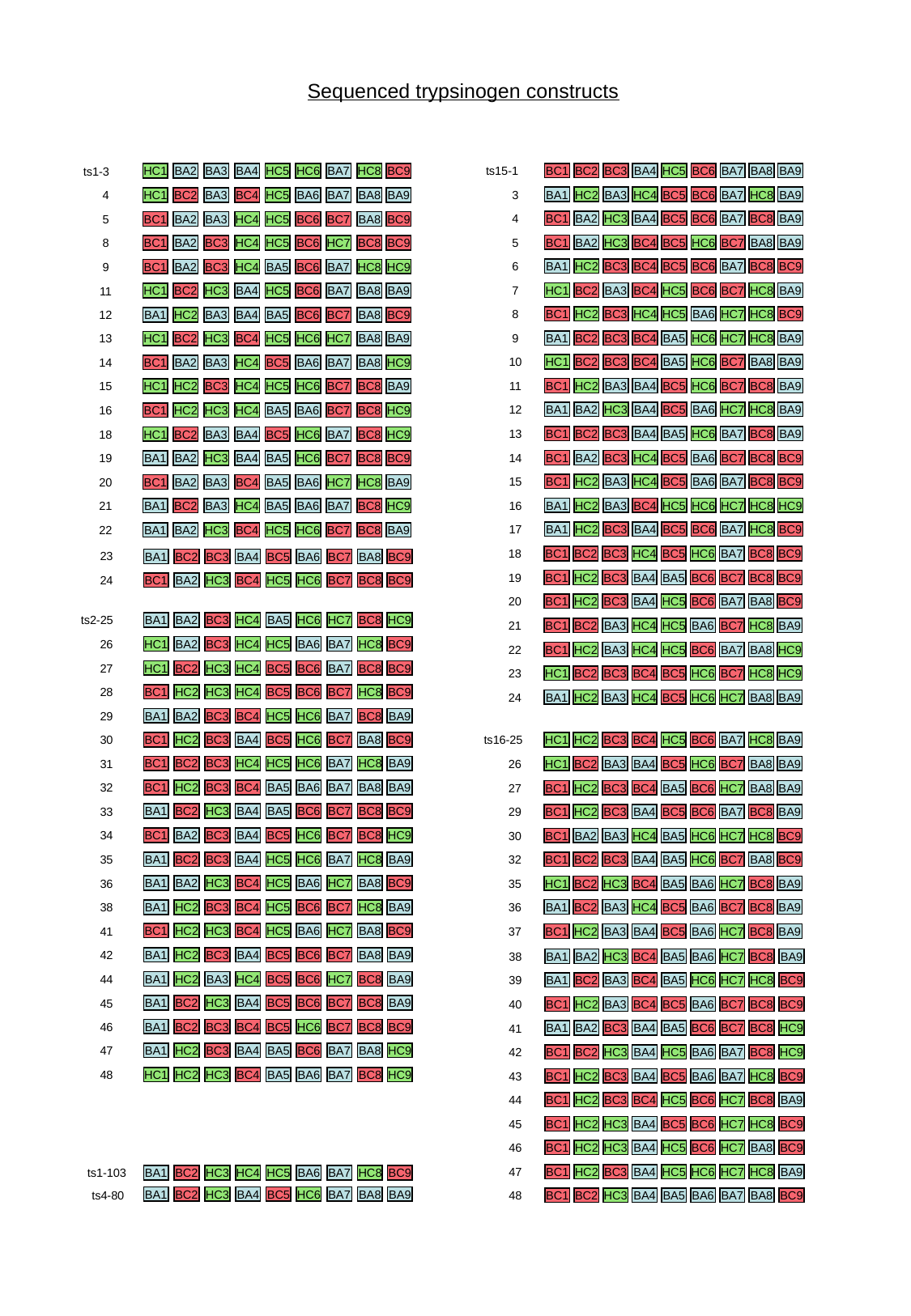

Sequenced trypsinogen constructs
ts15-1
BC1
BC2
BC3
BA4
HC5
BC6
BA7
BA8
BA9
ts1-3
HC1
BA2
BA3
BA4
HC5
HC6
BA7
HC8
BC9
3
BA1
HC2
BA3
HC4
BC5
BC6
BA7
HC8
BA9
4
HC1
BC2
BA3
BC4
HC5
BA6
BA7
BA8
BA9
4
BC1
BA2
HC3
BA4
BC5
BC6
BA7
BC8
BA9
5
BC1
BA2
BA3
HC4
HC5
BC6
BC7
BA8
BC9
5
BC1
BA2
HC3
BC4
BC5
HC6
BC7
BA8
BA9
8
BC1
BA2
BC3
HC4
HC5
BC6
HC7
BC8
BC9
6
BA1
HC2
BC3
BC4
BC5
BC6
BA7
BC8
BC9
9
BC1
BA2
BC3
HC4
BA5
BC6
BA7
HC8
HC9
7
HC1
BC2
BA3
BC4
HC5
BC6
BC7
HC8
BA9
11
HC1
BC2
HC3
BA4
HC5
BC6
BA7
BA8
BA9
8
BC1
HC2
BC3
HC4
HC5
BA6
HC7
HC8
BC9
12
BA1
HC2
BA3
BA4
BA5
BC6
BC7
BA8
BC9
9
BA1
BC2
BC3
BC4
BA5
HC6
HC7
HC8
BA9
13
HC1
BC2
HC3
BC4
HC5
HC6
HC7
BA8
BA9
10
HC1
BC2
BC3
BC4
BA5
HC6
BC7
BA8
BA9
14
BC1
BA2
BA3
HC4
BC5
BA6
BA7
BA8
HC9
11
BC1
HC2
BA3
BA4
BC5
HC6
BC7
BC8
BA9
15
HC1
HC2
BC3
HC4
HC5
HC6
BC7
BC8
BA9
12
BA1
BA2
HC3
BA4
BC5
BA6
HC7
HC8
BA9
16
BC1
HC2
HC3
HC4
BA5
BA6
BC7
BC8
HC9
13
BC1
BC2
BC3
BA4
BA5
HC6
BA7
BC8
BA9
18
HC1
BC2
BA3
BA4
BC5
HC6
BA7
BC8
HC9
14
BC1
BA2
BC3
HC4
BC5
BA6
BC7
BC8
BC9
19
BA1
BA2
HC3
BA4
BA5
HC6
BC7
BC8
BC9
15
BC1
HC2
BA3
HC4
BC5
BA6
BA7
BC8
BC9
20
BC1
BA2
BA3
BC4
BA5
BA6
HC7
HC8
BA9
16
BA1
HC2
BA3
BC4
HC5
HC6
HC7
HC8
HC9
21
BA1
BC2
BA3
HC4
BA5
BA6
BA7
BC8
HC9
17
BA1
HC2
BC3
BA4
BC5
BC6
BA7
HC8
BC9
22
BA1
BA2
HC3
BC4
HC5
HC6
BC7
BC8
BA9
18
BC1
BC2
BC3
HC4
BC5
HC6
BA7
BC8
BC9
23
BA1
BC2
BC3
BA4
BC5
BA6
BC7
BA8
BC9
19
BC1
HC2
BC3
BA4
BA5
BC6
BC7
BC8
BC9
24
BC1
BA2
HC3
BC4
HC5
HC6
BC7
BC8
BC9
20
BC1
HC2
BC3
BA4
HC5
BC6
BA7
BA8
BC9
ts2-25
BA1
BA2
BC3
HC4
BA5
HC6
HC7
BC8
HC9
BC1
BC2
BA3
HC4
HC5
BA6
BC7
HC8
BA9
21
26
HC1
BA2
BC3
HC4
HC5
BA6
BA7
HC8
BC9
22
BC1
HC2
BA3
HC4
HC5
BC6
BA7
BA8
HC9
27
HC1
BC2
HC3
HC4
BC5
BC6
BA7
BC8
BC9
23
HC1
BC2
BC3
BC4
BC5
HC6
BC7
HC8
HC9
28
BC1
HC2
HC3
HC4
BC5
BC6
BC7
HC8
BC9
24
BA1
HC2
BA3
HC4
BC5
HC6
HC7
BA8
BA9
29
BA1
BA2
BC3
BC4
HC5
HC6
BA7
BC8
BA9
30
BC1
HC2
BC3
BA4
BC5
HC6
BC7
BA8
BC9
ts16-25
HC1
HC2
BC3
BC4
HC5
BC6
BA7
HC8
BA9
31
BC1
BC2
BC3
HC4
HC5
HC6
BA7
HC8
BA9
26
HC1
BC2
BA3
BA4
BC5
HC6
BC7
BA8
BA9
32
BC1
HC2
BC3
BC4
BA5
BA6
BA7
BA8
BA9
27
BC1
HC2
BC3
BC4
BA5
BC6
HC7
BA8
BA9
33
BA1
BC2
HC3
BA4
BA5
BC6
BC7
BC8
BC9
29
BC1
HC2
BC3
BA4
BC5
BC6
BA7
BC8
BA9
34
BC1
BA2
BC3
BA4
BC5
HC6
BC7
BC8
HC9
30
BC1
BA2
BA3
HC4
BA5
HC6
HC7
HC8
BC9
35
BA1
BC2
BC3
BA4
HC5
HC6
BA7
HC8
BA9
32
BC1
BC2
BC3
BA4
BA5
HC6
BC7
BA8
BC9
36
BA1
BA2
HC3
BC4
HC5
BA6
HC7
BA8
BC9
35
HC1
BC2
HC3
BC4
BA5
BA6
HC7
BC8
BA9
36
BA1
BC2
BA3
HC4
BC5
BA6
BC7
BC8
BA9
38
BA1
HC2
BC3
BC4
HC5
BC6
BC7
HC8
BA9
41
BC1
HC2
HC3
BC4
HC5
BA6
HC7
BA8
BC9
37
BC1
HC2
BA3
BA4
BC5
BA6
HC7
BC8
BA9
42
BA1
HC2
BC3
BA4
BC5
BC6
BC7
BA8
BA9
38
BA1
BA2
HC3
BC4
BA5
BA6
HC7
BC8
BA9
44
BA1
HC2
BA3
HC4
BC5
BC6
HC7
BC8
BA9
39
BA1
BC2
BA3
BC4
BA5
HC6
HC7
HC8
BC9
45
BA1
BC2
HC3
BA4
BC5
BC6
BC7
BC8
BA9
40
BC1
HC2
BA3
BC4
BC5
BA6
BC7
BC8
BC9
46
BA1
BC2
BC3
BC4
BC5
HC6
BC7
BC8
BC9
41
BA1
BA2
BC3
BA4
BA5
BC6
BC7
BC8
HC9
47
BA1
HC2
BC3
BA4
BA5
BC6
BA7
BA8
HC9
42
BC1
BC2
HC3
BA4
HC5
BA6
BA7
BC8
HC9
48
HC1
HC2
HC3
BC4
BA5
BA6
BA7
BC8
HC9
43
BC1
HC2
BC3
BA4
BC5
BA6
BA7
HC8
BC9
44
BC1
HC2
BC3
BC4
HC5
BC6
HC7
BC8
BA9
45
BC1
HC2
HC3
BA4
BC5
BC6
HC7
HC8
BC9
46
BC1
HC2
HC3
BA4
HC5
BC6
HC7
BA8
BC9
47
BC1
HC2
BC3
BA4
HC5
HC6
HC7
HC8
BA9
ts1-103
BA1
BC2
HC3
HC4
HC5
BA6
BA7
HC8
BC9
BA1
BC2
HC3
BA4
BC5
HC6
BA7
BA8
BA9
ts4-80
48
BC1
BC2
HC3
BA4
BA5
BA6
BA7
BA8
BC9
