# Supplementary material for: Golden Gate Shuffling: A One-Pot DNA Shuffling Method Based on Type IIs Restriction Enzymes
Source: PLoS One. 2009 May 14;4(5):e5553. doi: 10.1371/journal.pone.0005553 (PMC2677662; doi:10.1371/journal.pone.0005553)
Supplement: Figure S4 — Structure of incorrect trypsinogen constructs obtained with the second set of modules. This figure shows the structure of incorrect trypsinogen constructs obtained with the second set of modules (mod2), and proposes models to explain their formation. (0.11 MB PPT) [file pone.0005553.s004.ppt]

## Slide 1
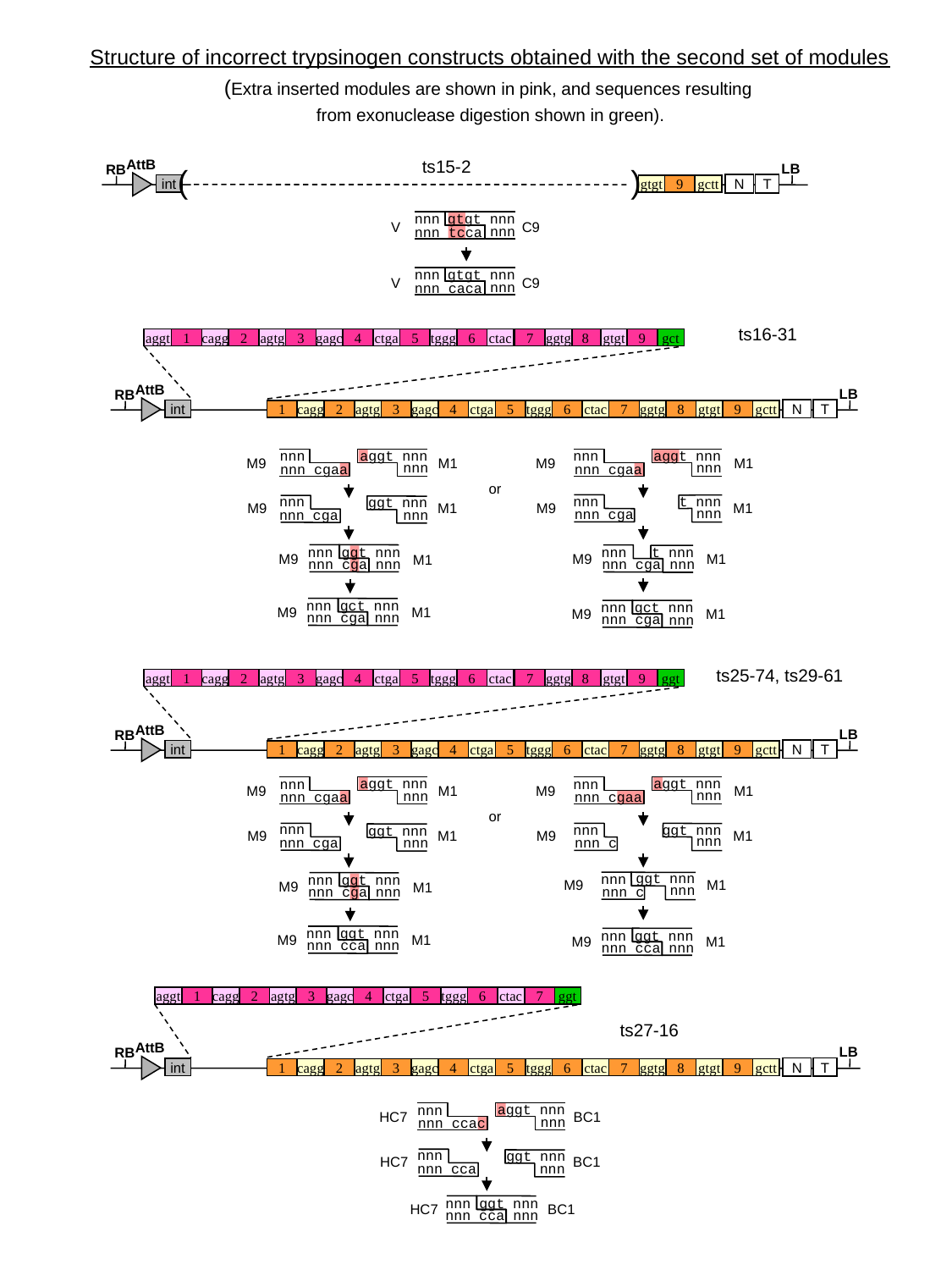

Structure of incorrect trypsinogen constructs obtained with the second set of modules (Extra inserted modules are shown in pink, and sequences resulting
from exonuclease digestion shown in green).
ts15-2
AttB
LB
RB
( )
N
T
int
gtgt
9
gctt
gtgt nnn
nnn
V
C9
nnn
nnn tcca
gtgt nnn
nnn
V
C9
nnn
nnn caca
ts16-31
aggt
1
cagg
2
agtg
3
gagc
4
ctga
5
tggg
6
ctac
7
ggtg
7
ggtg
8
gtgt
9
gct
AttB
LB
RB
N
T
int
1
cagg
2
agtg
3
gagc
4
ctga
5
tggg
6
ctac
7
ggtg
8
gtgt
9
gctt
aggt nnn
aggt nnn
nnn
nnn
M9
M1
M9
M1
nnn
nnn
nnn cgaa
nnn cgaa
or
t nnn
nnn
nnn
ggt nnn
M9
M1
M9
M1
nnn
nnn cga
nnn
nnn cga
nnn
ggt nnn
nnn
t nnn
M9
M1
nnn cga
nnn
M9
M1
nnn cga
nnn
nnn
gct nnn
nnn
gct nnn
M9
M1
M9
M1
nnn cga
nnn
nnn cga
nnn
ts25-74, ts29-61
aggt
1
cagg
2
agtg
3
gagc
4
ctga
5
tggg
6
ctac
7
ggtg
7
ggtg
8
gtgt
9
ggt
AttB
LB
RB
N
T
int
1
cagg
2
agtg
3
gagc
4
ctga
5
tggg
6
ctac
7
ggtg
8
gtgt
9
gctt
aggt nnn
aggt nnn
nnn
nnn
M9
M1
M9
M1
nnn
nnn
nnn cgaa
nnn cgaa
or
nnn
ggt nnn
nnn
ggt nnn
M9
M1
M9
M1
nnn
nnn
nnn cga
nnn c
ggt nnn
nnn
nnn
ggt nnn
M9
M1
M9
M1
nnn
nnn cga
nnn
nnn c
nnn
ggt nnn
nnn
ggt nnn
M9
M1
M9
M1
nnn cca
nnn
nnn cca
nnn
aggt
1
cagg
2
agtg
3
gagc
4
ctga
5
tggg
6
ctac
7
ggt
ts27-16
AttB
LB
RB
N
T
int
1
cagg
2
agtg
3
gagc
4
ctga
5
tggg
6
ctac
7
ggtg
8
gtgt
9
gctt
aggt nnn
nnn
HC7
BC1
nnn
nnn ccac
nnn
ggt nnn
HC7
BC1
nnn
nnn cca
nnn
ggt nnn
HC7
BC1
nnn cca
nnn
